# The short chitooligosaccharide CO4 inhibits chitin-triggered immunity in grapevine and promotes the infection by *Botrytis cinerea* but not *Plasmopara viticola*

**DOI:** 10.1093/jxb/eraf247

**Published:** 2025-06-04

**Authors:** Tania Marzari, Jérémy Villette, Thibault Roudaire, Karine Palavioux, Daphnée Brulé, Agnès Klinguer, Marie-Claire Héloir, Mathieu Gayral, Benoit Poinssot

**Affiliations:** Université Bourgogne Europe, Institut Agro, INRAE, Agroécologie, Dijon, France; Université Bourgogne Europe, Institut Agro, INRAE, Agroécologie, Dijon, France; Université Bourgogne Europe, Institut Agro, INRAE, Agroécologie, Dijon, France; Laboratoire des Interactions Plantes Microbes-Environnement (LIPME), CNRS, INRAE, Université de Toulouse, Castanet-Tolosan, Toulouse, France; Université Bourgogne Europe, Institut Agro, INRAE, Agroécologie, Dijon, France; Université Bourgogne Europe, Institut Agro, INRAE, Agroécologie, Dijon, France; Université Bourgogne Europe, Institut Agro, INRAE, Agroécologie, Dijon, France; Université Bourgogne Europe, Institut Agro, INRAE, Agroécologie, Dijon, France; Université Bourgogne Europe, Institut Agro, INRAE, Agroécologie, Dijon, France; Université Bourgogne Europe, Institut Agro, INRAE, Agroécologie, Dijon, France; University of Ghent, Belgium

**Keywords:** CO4, defense genes, immune responses, inhibition, LCOs, MAMPs, MAP kinases, *Vitis vinifera*

## Abstract

Plants have developed strategies to detect different microorganisms and specifically modulate their immune responses. A primary recognition involves the perception of highly conserved molecular signatures, also known as microbe-associated molecular patterns (MAMPs). Among them, chitin, the main component of the fungal cell wall, is well known to be particularly active in triggering immunity in many plant species, including grapevine. While chitin is a well-known elicitor of plant defenses, other MAMPs such as short chitooligosaccharides [e.g. chitotetraose (CO4)] and lipo-chitooligosaccharides (LCOs) have been described to promote symbiotic interactions and inhibit plant immunity in several plant species. Here, we analyzed the molecular signaling triggered by these MAMPs in grapevine, focusing on two key immune responses: phosphorylation of mitogen-activated protein kinases (MAPKs) and expression of defense genes. Our results revealed that CO4 is the most active MAMP to inhibit some immune responses normally triggered by chitin. In addition, CO4 pre-treatment of grapevine leaves resulted in the repression of immune responses and increased susceptibility to the fungal pathogen *Botrytis cinerea* while showing no effect on *Plasmopara viticola* infection. These results suggest that grapevine can regulate its immune signaling pathways differently to either block or promote microbial colonization, depending on the MAMP perceived.

## Teaser

*Botrytis cinerea* infection is promoted by chitotetraose treatment, which plays a role as a negative regulator of grapevine immunity, inhibiting MAPK phosphorylation and defense-related gene expression in both roots and leaves.

## Graphical Abstract

**Graphical Abstract:**
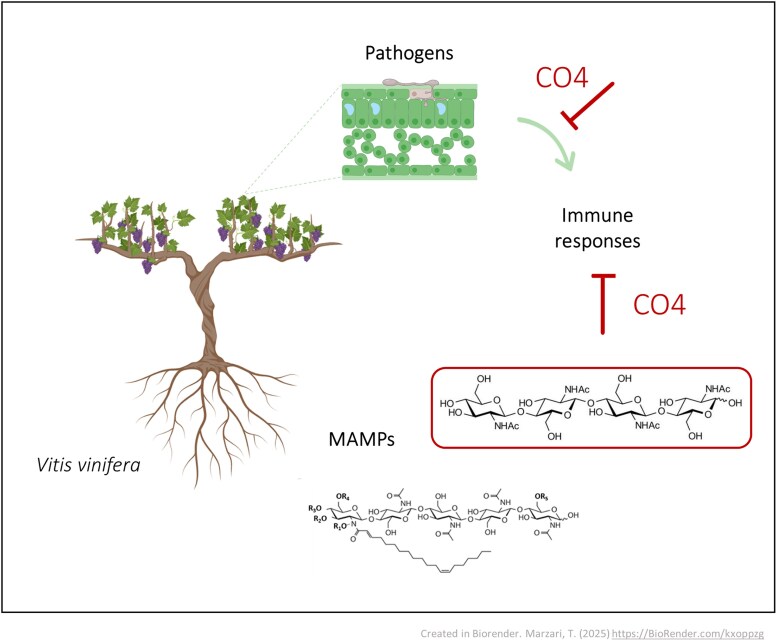

## Introduction

Plant–microbe interactions are an essential aspect of plant ecology and physiology, playing crucial roles in plant growth, health, and survival. In nature, plants are constantly exposed to a myriad of microorganisms spanning from mutualistic beneficial microorganisms to pathogenic ones. At the core of these interactions lies a sophisticated network of signaling pathways and molecular exchanges that determine whether a microbe will become a beneficial partner, a neutral bystander, or a detrimental pathogen to the plant host. This process mainly relies on the perception of a specific class of molecules called microbe-associated molecular patterns (MAMPs) which are essential structures for the microbes and are for that reason conserved among pathogens, non-pathogenic microorganisms, and saprophytic microorganisms (Newman *et al.*, 2013; Rush *et al.*, 2020). Plants deploy membrane-localized pattern recognition receptors (PRRs) to distinguish microbes by recognizing the MAMPs perceived, which trigger a specific signaling pathway (Zipfel and Oldroyd, 2017; Rey and Jacquet, 2018; Bentham *et al.*, 2020).

Chitin oligomers (COs) are among the most widespread and active fungal MAMPs, inducing a large set of molecular responses based on their nature and degree of polymerization (DP). COs are polymers of β-1,4-linked *N*-acetylglucosamine (GlcNAc) of different lengths derived from chitin, a polymer commonly found in the fungal cell walls as well as in the shells of arthropod exoskeletons and nematodes (Khokhani *et al.*, 2021). In *Vitis vinifera*, long-chain COs with a DP ≥6 are particularly active in promoting immune signaling events such as the activation of mitogen-activated protein kinases (MAPKs) and the expression of defense-related genes involved in the biosynthesis of phenylpropanoids and pathogenesis-related (PR) proteins (Brulé *et al.*, 2019; Roudaire *et al.*, 2023). In contrast, short-chain COs, such as chitotetraose (CO4), are known to be involved in the first steps of symbiotic interactions between plant and arbuscular mycorrhizal fungi (AMF) in many plant species including rice, barley, and several legumes (Maillet *et al.*, 2011; Genre *et al.*, 2013; Camps *et al.*, 2015; Sun *et al.*, 2015). In addition to CO4, the lipo-chitooligosaccharides (LCOs), which are composed of four or five GlcNAc units (DP ≤5), *N*-acylated by a fatty acid on the non-reducing terminal sugar and enriched by different chemical substitutions on the chitin backbone (Liang *et al.*, 2014; Rush *et al.*, 2020), are also widely considered as symbiotic MAMPs or symbiotic factors during arbuscular mycorrhiza symbiosis or rhizobium–legume symbiosis (Maillet *et al.*, 2011; Kamel *et al.*, 2017; Quilbé *et al.*, 2022). Whether with pathogens or mutualists, plant–microorganism interactions are the result of a fine modulation of plant immunity which is driven by MAMP perception and is first translated in the activation of MAMP-triggered immunity (MTI) (Newman *et al.*, 2013). Nevertheless, during beneficial symbiotic interactions, they also assist in the inhibition of the same MTI. Indeed, symbiotic microorganisms must evade plant defenses in order to colonize their host plant and improve their fitness. Several studies have shown that initiation of mutualistic symbiosis may involve inputs from overlapping immunity and symbiosis pathways, showing an early activation and later inhibition of plant defense responses (Lohar *et al.*, 2006; Campos-Soriano *et al.*, 2012). In *Medicago truncatula* and *Lotus japonicus* roots, LCO pre-treatment before chitin or flagellin elicitation led to a decreased level of MAPK phosphorylation and of defense-related genes expression (Lopez-Gomez *et al.*, 2012; Feng *et al.*, 2019). Similarly, in rice, CO4 had the same inhibitory effect on different defense responses, confirming a fine modulation of plant immunity (Sun *et al.*, 2015; Zhang *et al.*, 2021). Interestingly, the suppression of immune signaling events by symbiotic factors is also present in *Arabidopsis thaliana*, even though this plant species does not form root nodule or arbuscular mycorrhizal symbioses (Liang *et al.*, 2013; Zipfel and Oldroyd, 2017). It is possible that the production of LCOs and short-chain COs may be an ancient molecular mechanism which evolved in the first place by microorganisms to support a pathogenic role that has been adapted later to support symbiotic interactions (Liang *et al.*, 2014).

As for most plants, *Vitis vinifera* also copes with different microorganisms, whether mutualistic or pathogenic. In the latter category, we find the causal agents of some of the most critical diseases, including the necrotrophic agent of gray mold, *Botrytis cinerea*, the obligate biotrophic oomycete agent of downy mildew, *Plasmopora viticola*, and the obligate fungal pathogen causing grape powdery mildew, *Erysiphe necator*. These pathogens cause significant losses in terms of quantity and quality during both pre- and post-harvest periods (Armijo *et al.*, 2016; Adrian *et al.*, 2024). However, despite grapevine immune responses having been extensively studied in response to elicitors or pathogens, our knowledge about the inhibition of grapevine immunity caused by some MAMPs and their role during plant–pathogen interactions is still unclear.

In this work, we aimed to characterize the effect of different MAMPs on grapevine immunity, focusing on different organs such as roots and leaves. We showed that CO4 was the most active MAMP involved in the inhibition of grapevine immunity regardless of its concentration and of the organ treated. In addition, we demonstrated that CO4 cannot be considered just as a symbiotic molecule but rather it appears that short COs could also be considered as a signal used by some pathogens to overcome plant immunity in order to promote pathogen spreading during the infection process.

## Materials and methods

### Plant materials

*Vitis vinifera* cv Marselan (*V. vinifera* cv. Cabernet Sauvignon*×V. vinifera* cv. Grenache noir) cell culture was obtained from callus initiated from petioles of grapevine leaves and cultivated in Nitsch–Nitsch medium (Nitsch and Nitsch, 1969) supplemented with 1 g l^–1^ casein hydrolysate, 400 μg l^–1^ 1-naphthaleneacetic acid, and 40 μg l^–1^ 6-benzylaminopurine. This cell suspension was cultivated under continuous light (cool-white fluorescent tubes 3350L) with continuous shaking (120 rpm at 24 °C) and subcultured every 7 d by transferring 20 ml of the cell suspension into 100 ml of a new culture medium. For all experiments, a 7-day-old culture was diluted twice with fresh medium 1 d before use. Grapevine *in vitro* plantlets (*V. vinifera* cv Marselan) were kindly provided by the Institut universitaire de la vigne et du vin Jules Guyot located in Dijon, France. *In vitro* plantlets were grown on 15 ml of modified 7.5 g l^–1^ agar Murashige and Skoog (MS) in glass tubes (2 cm diameter, 15 cm height) and subcultured every 2 months. They were cultured in a growth chamber at 24±2 °C, under cool-white fluorescent tubes (3350L) with a 16 h day/8 h dark photoperiod.

*Vitis vinifera* cv Marselan herbaceous cuttings were grown in a greenhouse in individual pots (8 cm×8 cm×8 cm) containing a 7:3 (v/v) mixture of peat and perlite at 23 °C/15 °C (day/night) with a 16 h photoperiod. Plants were irrigated with a balanced nutrient solution (N:P:K, 10-10-10; Plantin, France) and were used for experiments once they had developed 5–7 fully expanded leaves. Both grapevine cell culture and *in vitro* plantlets were used for MAPK quantification and gene expression; grapevine cuttings were used for the infection assays with pathogens and for MAPK quantification and gene expression on leaves.

### Elicitors

Highly purified chitin with a DP of 6 (CH6, GLU436) and CO4 (GLU434) of DP4 were provided by Elicityl, Crolles, France. They were extracted from shellfish exoskeletons, hydrolyzed, purified by chromatography, and their final DP and acetylation degree were verified by ^1^H-NMR analysis. Three major LCOs were used in this study. LCO IV C18:1 and LCO IV C18:1S were provided by Dr Sébastien Fort (CERMAV, Grenoble), while LCO V 18:1 Fuc/MeFuc was provided by Dr Benoit Lefebvre (Laboratoire des interactions plantes-microbes-environnement, Toulouse). Due to the insolubility in water of sulfated LCOs, all of them were dissolved in 100% DMSO at a concentration of 10 mM for LCOs IV and 1 mM for LCO V, and stored at −20 °C. The CH6 and CO4 chitooligomers were dissolved in ultrapure water just prior to use, or in DMSO if they were used in parallel with LCO treatments to overcome solvent bias. Final experiments were carried out at a final concentration of 80 µM and 1 µM for chitin DP6, 120, 12, 1.2, and 0.1 µM for CO4, and 0.1 µM for LCOs.

### Phosphorylation of MAPKs

Grapevine cells, roots, and leaves of *in vitro* grapevine plantlets were harvested 10 min after CH6 treatment and immediately frozen in liquid nitrogen as in previous work (Brulé *et al.*, 2019) . For the inhibition assay, cell suspensions were pre-treated with either DMSO or LCOs (0.1 µM) or CO4 (ranging from 0.1 µM to 120 µM) for 30 min before being elicited by CH6 (1 µM or 80 µM) as previously described by Feng *et al.* (2019). *In vitro* plantlet roots and leaves were placed in 5 ml of water which was later replaced by the same volume of CO4 (120 µM) for 30 min or 48 h before being elicited by 5 ml of CH6 (80 µM). For cell suspension, proteins were extracted using an extraction buffer containing 50 mM HEPES (pH 7.5), 5 mM EGTA (pH 8.1), 5 mM EDTA, 1 mM Na_3_VO_4_, 50 mM β-glycerophosphate, 10 mM NaF, 1 mM phenylmethylsulfonyl fluoride, 5 mM DTT, and 1× complete™ EDTA-free Protease Inhibitor Cocktail (Roche). For roots and leaves of *in vitro* plantlets, tissues were ground in liquid nitrogen, and 100 mg of powder was used for protein extraction utilizing the TriReagent® protocol (Trizol, Sigma-Aldrich) with some modification. A 250 μl aliquot of TriReagent® was added to each sample followed by 25 μl of bromo-3-chloropropane. After centrifugation (15 min, 12 000 *g*, 4 °C), 75 μl of ethanol were added to the solid part and centrifuged a second time. The supernatant, containing proteins, was transferred to a microtube with 1.2 ml of glacial acetone for protein precipitation carried out overnight at −20 °C. The precipitate was then washed with 80% acetone and dissolved in 100 μl of Laemmli denaturation buffer (125 mM Tris–HCl pH 6.8, 0.2% SDS, 80 mM DTT). The phosphorylation of MAPKs was detected after immunoblotting of the extracted proteins (20 μg) using an anti-phospho-42/44-ERK antibody (Cell Signaling). The revealing step was performed on an Amersham™ ImageQuant™ 800 (Cytiva) using ECL™ Prime as a western blotting detection reagent. Transfer quality and homogeneous loading were checked by Ponceau red staining. MAPK was measured by ImageQuant software, and the band intensity of western blots was normalized on the loading control values represented by Rubisco. The normalized relative intensity shown in the figures represents the results of at least three biological independent repetitions.

### Real-time quantitative reverse transcription–PCR

Grapevine cell suspension, and roots or leaves of *in vitro* plantlets were treated as for assessing MAPK activation and harvested 1, 3, 9, and/or 24 h after CH6 treatment. Four independent biological replicates (*n*=4) have been realized for each quantitative reverse transcription–PCR (RT–qPCR) analysis. For cell suspension, total plant RNAs were extracted using the SV Total RNA Isolation System kit (Promega) with DNase treatment according to the supplier’s instructions. Reverse transcription was performed on 1 μg of total RNA using the High-Capacity cDNA Reverse Transcription kit (Applied Biosystems). RNAs from 80 mg of grapevine roots or leaves were extracted using the Spectrum™ Plant Total RNA Kit (Sigma) by adding ∼10 mg of polyvinylpolypyrrolidone (PVPP) before the application of the lysis buffer. Reverse transcription was performed on 1 μg of total RNA using the SuperScript IV Reverse Transcriptase kit (Thermofisher). Real-time qPCR was performed with 10 ng of cDNA thus generated, diluted in a total volume of 5 μl of GoTaq® qPCR Master Mix (Promega) for cell suspension and ABsolute QPCR Mix SYBR Green low ROX (Thermofisher) for leaves or roots of *in vitro* plantlets. Amplification of cDNA was carried out in a ViiA™ 7 Real-Time PCR (Applied Biosystems) thermocycler according to the following program: 15 min denaturation and activation of DNA polymerase at 95 °C followed by 40 cycles of three stages with 15 s at 95 °C (denaturation), 30 s at 61 °C (primer hybridization), and 30 s at 72 °C (amplification). A final step consisting of 15 s at 95 °C, 1 min at 61 °C, then increasing by 0.05 °C s^–1^ until 95 °C was finally performed to obtain the melting curves and thus confirm that the primers were specific for the target genes. Expression data were analyzed from the average of the threshold cycle (CT) values taking into consideration the efficiency (E) of each reaction calculated by the LinRegPCR quantitative PCR data analysis program (Ruijter *et al.*, 2009). For each biological replicate, the mean of the data resulting from the technical duplicates for each gene of interest was normalized on two reference genes: *VvVSP54* (*Vitvi10g01135*) and *VvRPL18B* (*Vitvi05g00033*) for grapevine cell suspension, and *VvVATP16* (*Vitvi03g04022*) and *VvEF1α* (*Vitvi06g04109*) for grapevine *in vitro* roots and leaves (Terrier *et al.*, 2005; Gamm *et al.*, 2011). Statistically significant differences between means (*n*=4) of the different treatments using a Kruskal–Wallis multiple comparison test followed by a ‘Bonferroni test’ correction (*P*<0.05) are indicated with different letters. Concerning *E. necator* infection, the ratio between the *V. vinifera* reference gene CT (*VvEF1α*) and the *E. necator* reference gene CT (*VvEN*) (Dufour *et al.*, 2011) was calculated for pathogen quantification. The primers used can be found in Supplementary Table S1.

### Pathogen infection

For *B. cinerea* infection assays, 20 leaf discs (1.9 cm diameter) from the upper third leaves of adult plants were floated on either 120 µM CO4 or H_2_O (as a control) during 48 h. Next, leaf discs were placed on damp Whatmann paper to be inoculated on the upper side with 1000 conidia of the BMM strain (Zimmerli *et al.*, 2001) in a drop of 20 µl of 1/4-diluted potato dextrose broth (PDB). The inoculated leaf discs were placed in a plastic box maintained at 100% humidity under a 10/14 h day/night photoperiod at 20/18 °C. Disease intensity was assessed 3 days post-inoculation (dpi) by measuring the *B. cinerea* lesion diameter using image J software (https://imagej.net/ij/).

For *P. viticola* infection assays, 36 leaf discs (1.3 cm diameter) from the first developed leaf of adult plants were floated on either 120 µM CO4 or H_2_O (as a control) for 48 h. Leaf discs were next transferred onto damp Whatmann paper in a plastic box and sprayed on the lower side with a solution of 10^4^ sporangia ml^–1^  *P. viticola.* The box was maintained in 100% humidity in the dark for 24 h, then under a 10/14 h day/night cycle at 20/18 °C. Disease intensity was assessed at 7 dpi by measuring the sporulating area by image analysis using the Visilog 6.9 software (Kim Khiook *et al.*, 2013).

For *E. necator* infection, the second youngest leaf of nine grapevine cuttings was sprayed in a greenhouse with either water or 120 µM CO4 for 48 h. Previously treated leaves were then inoculated with a suspension of *E. necator* 48 h post-treatment, and transferred in a plastic box maintained in 100% relative humidity under a 10/14 h day/night cycle at 20/18 °C. Disease intensity was assessed at 12 dpi by evaluating symptom development on each leaf following the evaluation method developed by the International Organization of Vine and Wine (OIV 455-1).

### Statistical analyses

All statistical analyses were performed using R software (http://cran.r-project.org) with the Agricolae package, performing the Kruskal–Wallis multiple comparison test with Bonferroni or Benjamini–Hochberg test correction (*P*<0.05). Statistical analysis of the pathogen infection test was performed using Wilcoxon test followed by Bonferroni test correction (*P*<0.05).

## Results

### CO4 inhibits chitin-triggered immunity in grapevine cell suspension

LCOs and short COs are MAMPs commonly found in fungi and they can both inhibit immune signaling induced by chitin or the bacterial flagellin flg22 in different plant species (Liang *et al.*, 2013; Feng *et al.*, 2019). Here we explored the effect of these molecular patterns in grapevine (*V. vinifera*), an economically important crop. In this study, we used three different LCOs, either fucosylated/methyl fucosylated LCO V C18:1 purified from *Rhizophagus irregularis*, referred to as LCO V AMF, or LCO IV C18:1 S and LCO IV C18:1 synthesized in genetically modified bacteria as previously described (Maillet *et al.*, 2011). In addition, CO4 was also chosen since its role as an inhibitor was previously shown during the rice–*R. irregularis* interaction (Zhang *et al.*, 2021).

**Fig. 1.**
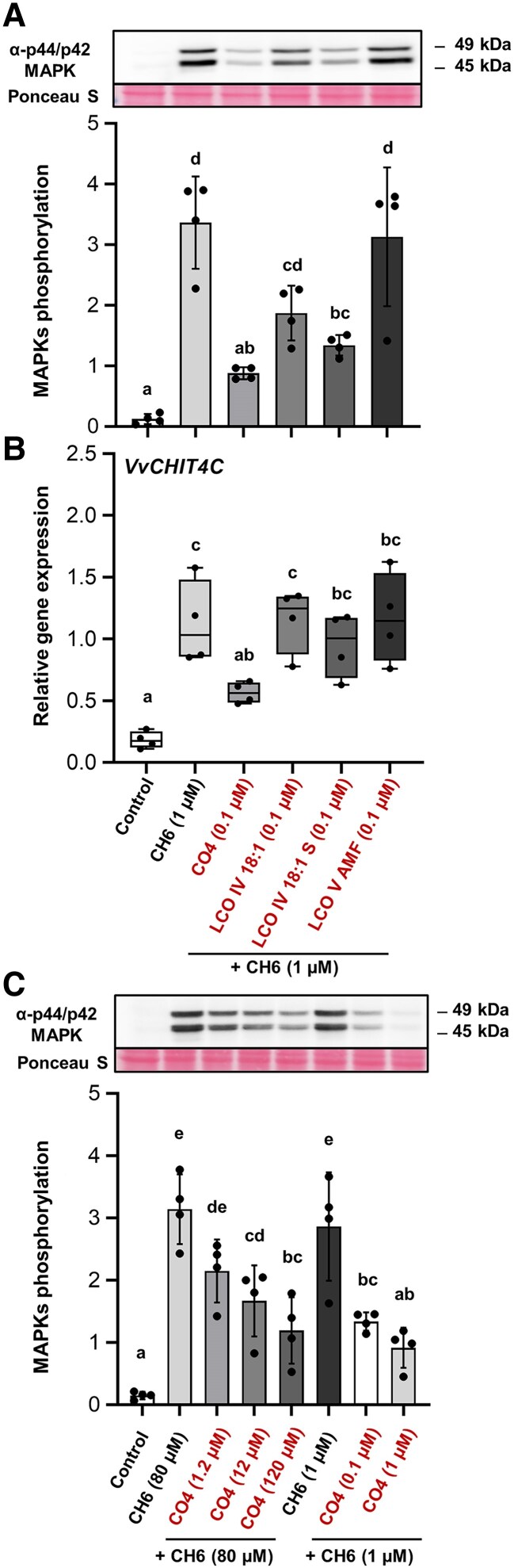
Treatment with the short chitooligosaccharide chitotetraose (CO4) reduces the chitin-triggered immunity in grapevine cell suspension. (A) Mitogen-activated protein kinase (MAPK) phosphorylation induced 10 min after treatment by 1 µM chitin hexamer (CH6) with or without a 30 min pre-treatment by 0.1 µM CO4, LCO IV 18:1, LCO IV 18:1 S, or LCO V AMF detected by immunoblotting with an antibody raised against the human phosphorylated MAPKs (p-ERK1/2). Equal protein loading was confirmed by Ponceau S red staining. The graph shows quantification of protein levels. Data are means ±SD from four independent biological repeats (*n*=4). Different letters indicate statistically significant differences between treatments using a Kruskal–Wallis multiple comparison test with a ‘Bonferroni test’ correction, (*P*<0.05). (B) Relative expression of the defense-related gene *VvCHIT4C* measured by qRT–PCR 1 h after chitin elicitation following a 30 min pre-treatment with CO4 or the different LCOs, as described in (A). Boxplots represent the distribution of four independent biological repeats (*n*=4). Means of technical duplicates [efficiency-weighted Cq(w) values] were normalized using mean Cq(w) data of two housekeeping genes (*VvVSP54* and *VvRPL18B*) before being analyzed. Different letters indicate statistically significant differences between treatments using a Kruskal–Wallis multiple comparison test followed by a ‘Bonferroni test’ correction (*P*<0.05). (C) Dose response inhibition of MAPK phosphorylation induced 10 min after treatment with chitin (CH6 at 80 µM or 1 µM) with or without a 30 min pre-treatment with CO4 (at 1.2, 12, 120 µM or 0.1–1 µM) detected by immunoblotting with an antibody raised against the human phosphorylated MAPKs (p-ERK1/2). Equal protein loading was confirmed by Ponceau S red staining. The graph shows quantification of protein levels. Data are means ±SD from four independent biological repeats (*n*=4). Different letters indicate statistically significant differences between treatments (Kruskal–Wallis multiple comparison test followed by ‘Benjamini–Hochberg test’ correction (*P*<0.05).

One of the first immune signaling events triggered after chitin treatment is the phosphorylation of MAPKs, which can be immunodetected with an anti-p44/42 MAPK antibody. To investigate whether the different LCOs and/or CO4 can suppress chitin-triggered immune responses, a grapevine cell suspension was pre-treated with 0.1 µM LCOs or CO4 for 30 min followed by 10 min elicitation with 1 µM CH6 before detecting the phosphorylation of MAPKs. CH6 elicitation without pre-treatment induced a rapid phosphorylation of two MAPKs with relative molecular masses of 45 kDa and 49 kDa which was not observed in DMSO control-treated cells (Fig. 1A), confirming previous results (Brulé *et al.*, 2019). Among all MAMPs used as pre-treatments, CO4 and LCO IV C18:1 S induced a significant inhibition of MAPK phosphorylation compared with CH6 elicitation alone (Fig. 1A). Of these, CO4 pre-treatment showed the greatest effect, decreasing the CH6-triggered MAPK phosphorylation by almost 66%. LCO derived from *R. irregularis* (LCO V AMF) did not have any effect, showing the same level of phosphorylation as CH6 alone. The pre-treatment with the synthesized LCO IV C18:1 showed a slight inhibition but this not statistically different from treatment with CH6, taken as the positive control (Fig. 1A). To further analyze the effect of these MAMPs on other immune responses, the expression of a defense gene encoding an acidic chitinase (*CHIT4C*) and known to be induced by different elicitors in grapevine was examined by RT–qPCR (Trdá *et al.*, 2014; Brulé *et al.*, 2019). As for MAPK phosphorylation, LCO V AMF and LCO IV C18:1 did not show any significant inhibitory effect after CH6 elicitation (Fig. 1B). Surprisingly, LCO IV C18:1 S did not show any significant inhibitory effect, in contrast to MAPK phosphorylation analysis. One hour after CH6 elicitation, only the CO4 pre-treatment was able to significantly decrease the expression level of *VvCHIT4C* (Fig. 1B). At 3 h post-elicitation, no significant inhibition was shown for the expression of the same gene after all the different treatments (Supplementary Fig. S1).

To validate that CO4 could inhibit grapevine immunity regardless of the concentration, different ratios between CO4 pre-treatment and CH6 elicitation were analyzed by MAPK phosphorylation immunoblotting. Among the physiological concentrations used in Fig. 1A, we used an equimolar ratio of CH6 and CO4 (1 µM), or an equimassic ratio at 0.1 mg ml^–1^ equivalent to 80 µM CH6 and 120 µM CO4. Additionally, we also tested increasing concentrations of CO4, ranging from 1.2 µM to 120 µM, with a fixed concentration of CH6 (80 µM). As expected, all ratios tested showed significant inhibition of MAPK phosphorylation, except for the lower CO4/CH6 ratio (CO4 1.2 µM/CH6 80 µM) (Fig. 1C). Taken together, all these results show that CO4 acts as an inhibitor of grapevine immunity.

### CO4 inhibits defense response in grapevine roots of *in vitro* plantlets

**Fig. 2.**
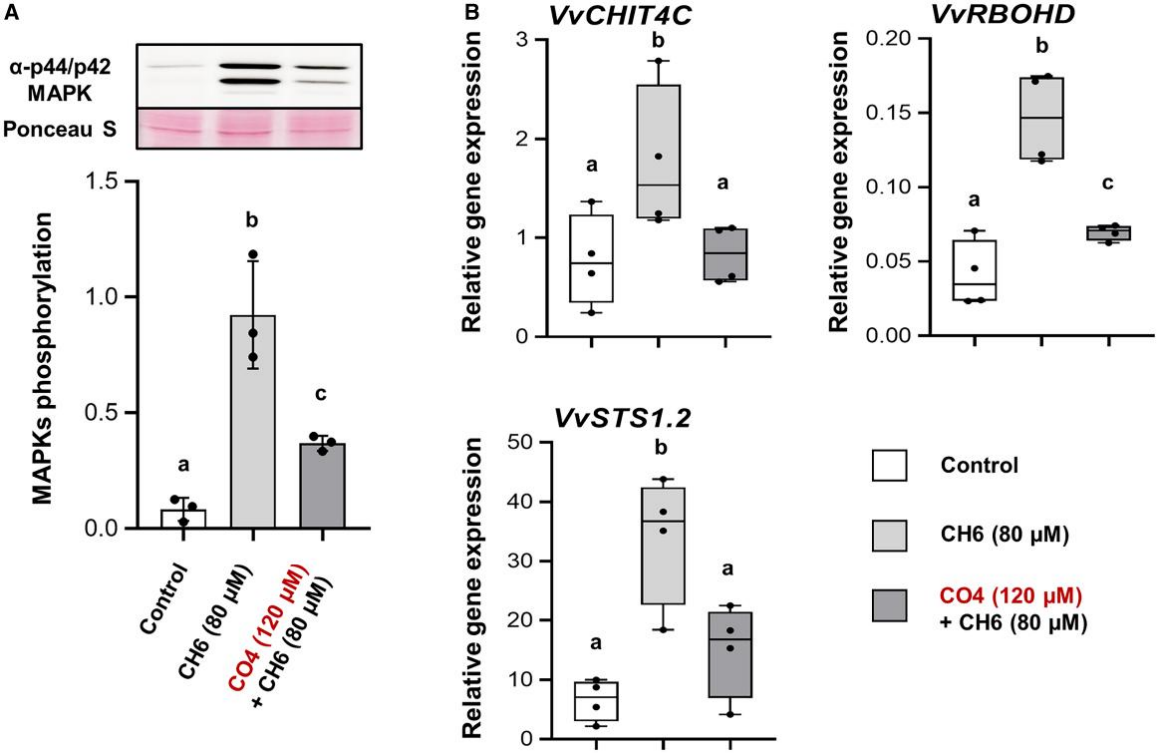
Chitotetraose (CO4) reduces chitin-triggered immunity in grapevine roots. (A) MAPK phosphorylation induced by 80 µM CH6 after 10 min with or without a 30 min pre-treatment by 120 µM CO4 and detected by immunoblotting with an antibody raised against the human phosphorylated MAPKs (p-ERK1/2). Equal protein loading was confirmed by Ponceau S red staining. The graph shows quantification of protein levels. Data are means ±SD from three independent biological repeats (*n*=3). Different letters indicate statistically significant differences between treatments using a Kruskal–Wallis multiple comparison test followed by ‘Benjamini–Hochberg test’ correction (*P*<0.05). (B) Relative expression of the defense-related genes *VvCHIT4C*, *VvRBOHD*, and *VvSTS1.2* measured by qRT–PCR 1 h after chitin elicitation (80 µM CH6) following a 30 min pre-treatment with CO4 (120 µM). Boxplots represent the distribution between four independent biological repeats (*n*=4). Means of technical duplicates [efficiency-weighted Cq(w) values] were normalized using mean Cq(w) data of two housekeeping genes (*VvVATP16* and *VvEF1α*) before being analyzed. Different letters indicate statistically significant differences between treatments using a Kruskal–Wallis multiple comparison test followed by ‘Benjamini–Hochberg test’ correction (*P*<0.05).

To further explore the role of CO4 in suppressing grapevine immunity, we decided to study its effect on roots which are the target of most mutualistic interactions. According to previous works (Brulé *et al.*, 2019; Roudaire *et al.*, 2023), we decided to use 0.1 mg/ml CH6 (equivalent to 80 µM) to elicit grapevine immune responses of *in vitro* grapevine plantlets. Previously, we also demonstrated that the equimassic ratio with 120 µM CO4 significantly reduced the elicitation triggered by 80 µM CH6 (Fig. 1C). As for grapevine cell suspension, CH6 treatment induced a strong phosphorylation of MAPKs in treated roots compared with control roots, and a 30 min CO4 pre-treatment significantly inhibits MAPK phosphorylation with an effective reduction by ∼50% when compared with CH6 treatment alone (Fig. 2A). To confirm the CO4 inhibitory effect, we also analyzed its effect on the expression of *VvCHIT4C* and two other genes involved in MAMP-triggered immunity: a stilbene synthase (*STS*) and a respiratory burst oxidase homolog D (*RBOHD*) (Kelloniemi *et al.*, 2015). CO4 pre-treatment was able to inhibit chitin-triggered elicitation of *VvCHIT4C*, as for grapevine cell suspension, but also of *VvSTS1.2* and *VvRBOHD* after 1 h of CH6 elicitation in grapevine roots of *in vitro* plantlets (Fig. 2B). In particular, the expression level of *VvCHIT4C* and *VvSTS1.2* was the same as that of the control, showing total inhibition. As for the cell suspension, no inhibition was shown after 3 h of CH6 elicitation (Supplementary Fig. S2A). In addition, we also checked the expression levels of various genes encoding enzymes of the phenylpropanoid pathway (*VvPAL* and *VvSTS1.2*), a lipoxygenase (*VvLOX9*), a reactive oxygen species (ROS)-related NADPH-oxidase (*VvRBOHD*), a protein related to the jasmonate pathway (*VvJAZ*), and the PR protein VvCHIT4C (Dufour *et al.*, 2016) at 1 h and 3 h post-elicitation. None of these genes was affected by CO4 pre-treatment (Supplementary Fig. S2B). These results show that CO4 acts as an inhibitor in grapevine roots, inducing a suppression of both MAPK phosphorylation and expression of some defense-related genes in the early immune response.

### CO4 inhibits chitin-triggered responses and promotes *Botrytis cinerea* infection in grapevine leaves

**Fig. 3.**
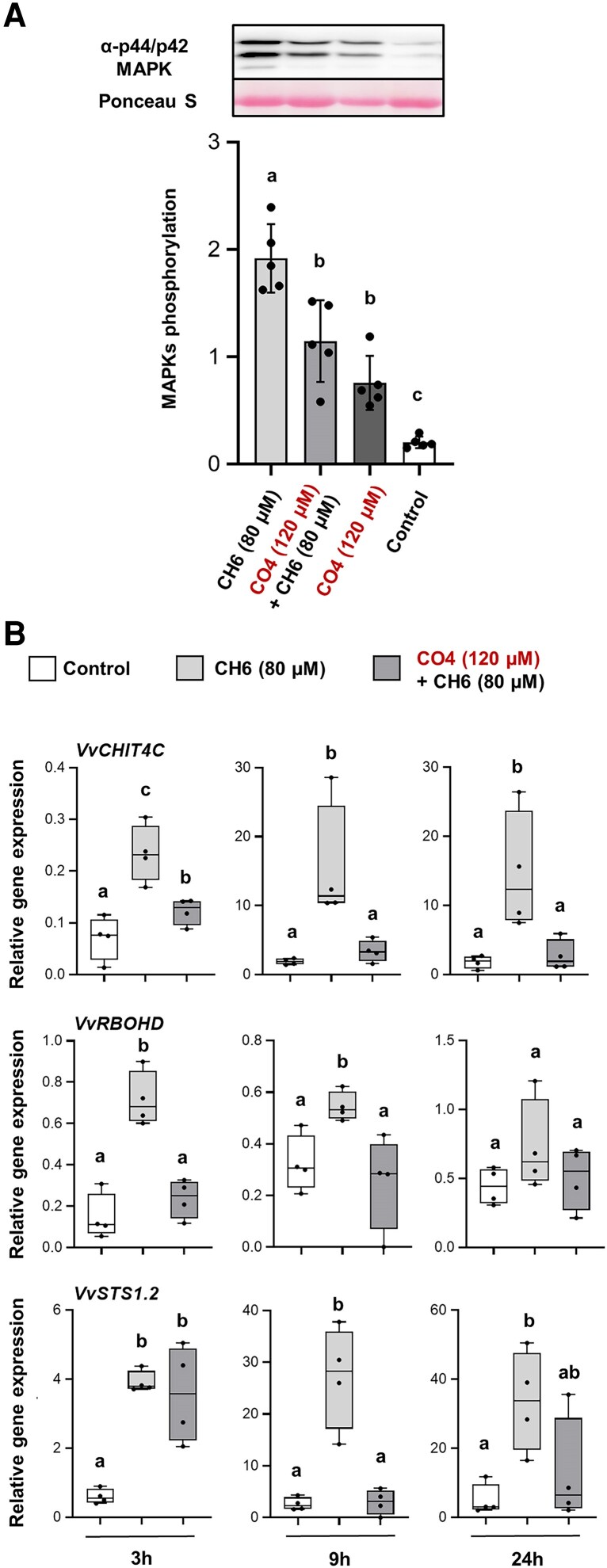
Chitotetraose (CO4) treatment reduces chitin-triggered immunity in grapevine leaves. (A) MAPK phosphorylation induced by 80 µM CH6 after 10 min with or without 48 h pre-treatment with 120 µM CO4 and obtained by immunoblotting with an antibody raised against the human MAPKs (p-ERK1/2). Equal protein loading was confirmed by Ponceau S red staining. The graph shows quantification of protein levels. Data are means ±SD from five independent biological repeats (*n*=5). Comparison test followed by ‘Bonferroni test’ correction (*P*<0.05). (B) Relative expression of the defense-related genes *VvCHIT4C*, *VvRBOHD*, and *VvSTS1.2* measured by qRT–PCR 3, 9, and 24 h after chitin elicitation (80 µM CH6) following a 48 h pre-treatment with CO4 (120 µM). Boxplots represent the distribution between four independent biological repeats (*n*=4). Means of technical duplicates [efficiency-weighted Cq(w) values] were normalized using mean Cq(w) data of two housekeeping genes (*VvVATP16* and *VvEF1α*) before being analyzed. Different letters indicate statistically significant differences between treatments using a Kruskal–Wallis multiple comparison test followed by ‘Benjamini–Hochberg test’ correction (*P*<0.05).

It was previously assumed that short-chain COs function as symbiotic MAMPs, mainly involved during beneficial interactions in Medicago and rice (Genre *et al.*, 2013; Zhang *et al.*, 2021; Volpe *et al.*, 2023). Interestingly, CO4 was also found to inhibit immunity in Arabidopsis, a plant species which does not interact either with rhizobium or with AMF (Liang *et al.*, 2013). We therefore decided to study the effect of CO4 pre-treatment on grapevine leaves from greenhouse cuttings in order to evaluate a putative organ-dependent signaling pathway. As for grapevine roots, we used 80 µM CH6 and 120 µM CO4. Surprisingly, CO4 alone showed a slight phosphorylation of MAPKs compared with the control (Fig. 3A). When CO4 was used as pre-treatment, we could still observe a strong inhibition of MAPK phosphorylation which decreased by almost 50% compared with CH6 elicitation (Fig. 3A). Additionally, CO4 pre-treatment led to a pronounced and prolonged suppression of *VvCHIT4C*, *VvRBOHD*, and *VvSTS1.2* expression following chitin elicitation in grapevine leaves (Fig. 3B). The inhibition persisted for up to 24 h post-elicitation, with *VvCHIT4C* showing a strong suppression maintained throughout this period. *VvRBOHD* expression remained reduced until 9 h post-elicitation, while *VvSTS1.2* showed decreased expression at both 9 h and 24 h post-elicitation (Fig. 3B). To further characterize the effect of CO4, we also analyzed the expression levels of various genes encoding enzymes of the phenylpropanoid pathway, PR proteins, a protein related to the jasmonate pathway, and a lipoxygenase. However, a significant effect of CO4 pre-treatment could not be observed at any time point for the different genes, except for *VvJAZ* whose CH6-triggered expression seems to be suppressed at 9 h and 24 h post-elicitation (Supplementary Fig. S3). On the whole, these results confirm that CO4 is a negative regulator of chitin-triggered immune responses in grapevine roots but also in leaves where the inhibition effect can last up to 24 h post-chitin elicitation.

### CO4 promotes *Botrytis cinerea* infection in grapevine leaves

**Fig. 4.**
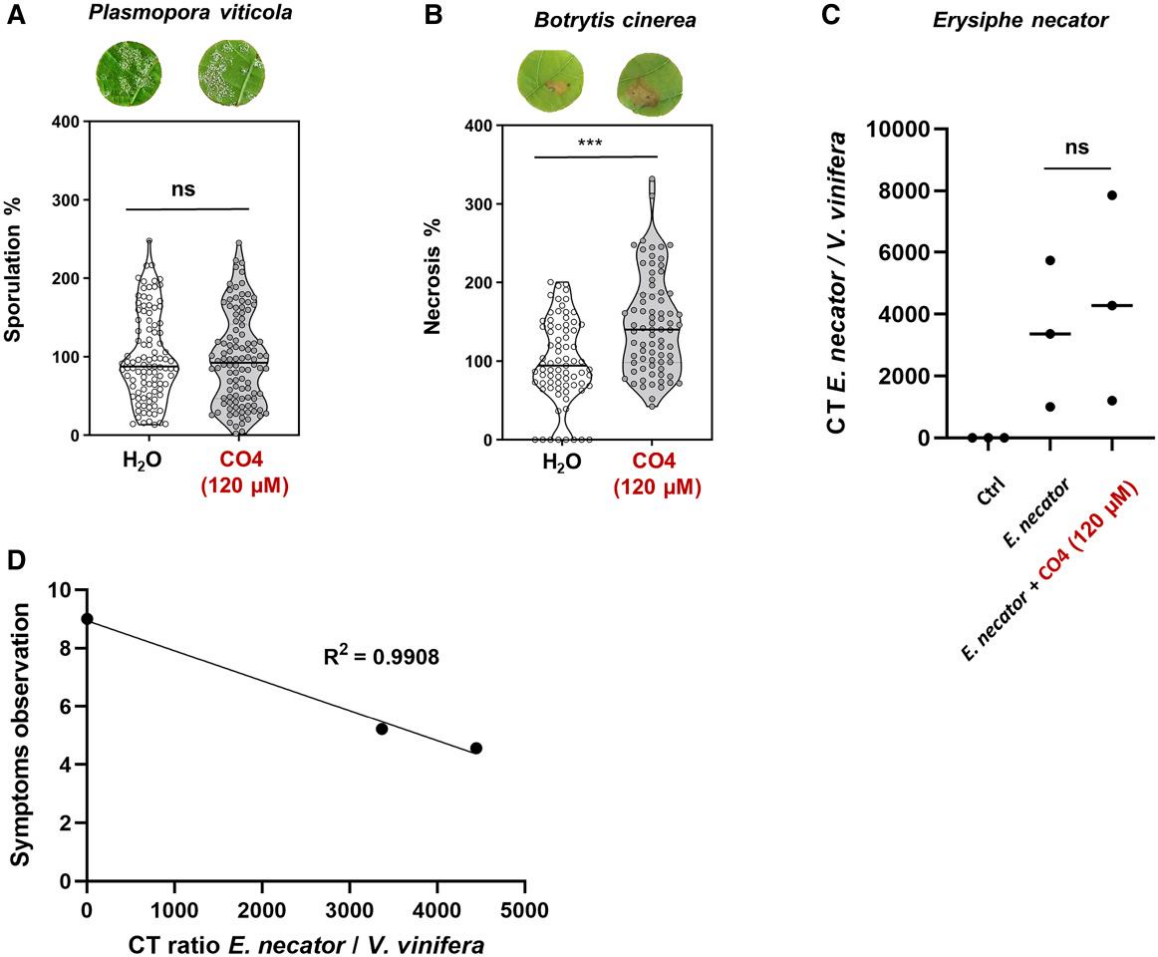
Chitotetraose (CO4) treatment promotes *B. cinerea* infection. (A) Development of *P. viticola* at 6 days post-inoculation (dpi) on grapevine leaf discs treated 48 h before with 120 µM CO4 and compared with control (H_2_O). The black line represents the mean percentage of each group. Dots represent the distribution of each value from six independent biological repeats (*n*=6) using at least 18 inoculated leaf discs from three different plants for each biological experiment (ns: not significant). (B) Development of *B. cinerea* at 3 dpi on grapevine leaf discs treated 48 h before with 120 µM CO4 and compared with control (H_2_O). Asterisks indicate statistically significant differences compared with the control using a Wilcoxon test followed by ‘Bonferroni test’ correction (*P*<0.05). The black line represents the mean percentage of each group. Dots represent the distribution of each value from four independent biological repeats (*n*=4). For each biological experiment, at least 18 leaf discs from three different plants were inoculated. (C) RT–qPCR determination of the CT ratio between the *E. necator* and the *V. vinifera* reference genes of three biological repeats (*n*=3) using at least three inoculated leaves for each biological experiment (ns: not significant). (D) Simple linear correlation realized (Graphpad Prism software) with *R*^2^=0.9908 showing negative correlation between RT–qPCR pathogen quantification and symptom observation using descriptors recommended by the OIV (OIV 455-1).

Since grapevine leaves are the target of numerous pathogens, we wondered if CO4 inhibition of plant immune responses could actively play a role during a direct interaction with a pathogen. To test this hypothesis, pathogenic assays were conducted where leaf discs and grapevine leaves pre-treated with CO4 were inoculated with either *B. cinerea*, *P. viticola*, or *E. necator.* The development of *P. viticola* or *B. cinerea* on *V. vinifera* leaf discs was monitored at 6 or 3 dpi, respectively, while the development of *E. necator* was assessed on detached leaves at 12 dpi. Surprisingly, we could not find any effect after inoculation with the oomycete *P. viticola*. Indeed, the analysis of *P. viticola* sporulation showed no significant difference between CO4-pre-treated plants and the control plants (Fig. 4A). On the other hand, CO4 pre-treatment resulted in a 50% increase of leaf necrosis caused by *B. cinerea* compared with non-pre-treated plants (Fig. 4B). For *E. necator* infection, a similar trend could be observed, with increased sporulation in plants pre-treated with CO4 (Fig. 4C). These pre-treated plants exhibited higher infection levels, as evidenced by both symptom observations using the OIV scale and pathogen quantification by RT–qPCR, which are strongly correlated (Fig. 4D; Supplementary Fig. S4). These results suggest that beyond *in vitro* CH6 elicitation, CO4 can also inhibit defense responses during a compatible plant–pathogen interaction, particularly with fungal pathogens whose cell wall is mainly composed of chitin.

## Discussion

The interaction between plants and microbes is a complex, dynamic, and continuous process that dates back to plant colonization on Earth. This rich environment led plants to develop strategies to detect and recognize all different microorganisms. Vice versa, microorganisms have also developed molecular mechanisms to counteract plant immunity (Ngou *et al.*, 2022). In the last decades, major findings on plant–microbe interactions have been identified. Nevertheless, many controversial questions remain about how plants differentiate pathogenic from mutualistic microorganisms and how plants regulate their immunity to adapt to the latter. Knowing the extreme diversity of interactions existing between plants and microorganisms, it is therefore not surprising that still today the way in which plants engage with beneficial microorganisms, while at the same time restricting pathogens, remains an open question (Harris *et al.*, 2020). Here, we describe how CO4 inhibits grapevine immunity, facilitating infection by the pathogenic fungus *B. cinerea*.

### CO4 is the most active tested MAMP to inhibit chitin-triggered immune responses in grapevine

One of the milestones in plant–microbe interactions is MAMP perception. MAMPs are found in many types of microorganisms, including bacteria, fungi, and viruses. They tend to be highly conserved, meaning that they are structurally similar across different species of microorganisms (Newman *et al.*, 2013). Chitin is constantly subject to the action of chitinolytic enzymes such as endochitinases which loosen the covalently linked chitin bonds to generate different fragments of chitin characterized by various lengths (Khokhani *et al.*, 2021). Notably, chitin oligomers of between six and eight molecules of GlcNAc are well known elicitors of defense responses (Miya *et al.*, 2007; Cao *et al.*, 2014; Hayafune *et al.*, 2014; Brulé *et al.*, 2019; Li *et al.*, 2020; Roudaire *et al.*, 2023). However, shorter chitin oligomers with a maximum DP of 4 or 5 (CO4 and LCOs) have been described mainly as symbiotic MAMPs. These specific MAMPs, called Myc and Nod factors, respectively, showed a larger role during symbiotic interactions during AMF and rhizobium symbiosis, with an important implication as inhibitors of plant immunity (Sun *et al.*, 2015; Feng *et al.*, 2019; Girardin *et al.*, 2019; Zhang *et al.*, 2021).

In grapevine, it has been demonstrated that chitin DP6 (CH6) triggers grapevine immune responses such as phosphorylation of MAPKs and the expression of defense genes notably encoding an acidic chitinase, a stilbene synthase, a phenylalanine ammonia lyase, and a respiratory burst oxidase homolog D (Brulé *et al.*, 2019). Here, we characterized the role of LCOs and CO4 in grapevine cell suspension, then focusing on different organs such as leaves and roots. Compared with three different LCOs (LCO IV C18:1, LCO IV C18:1 S, and LCO V 18:1 Fuc/MeFuc), we showed that CO4 is the most active MAMP for altering grapevine immune responses such as the chitin-induced MAPK phosphorylation and defense gene expression in cell suspension (Fig. 1A, B). We also confirmed that CO4 clearly inhibits chitin-triggered immunity in both grapevine roots (Fig. 2A, B) and leaves (Fig. 3A, B). COs were originally identified in nitrogen-fixing rhizobial bacteria (D’Haeze and Holsters, 2002) and more recently in symbiotic and pathogenic fungi (Rush *et al.*, 2020). Since grapevine is also capable of developing beneficial mutualistic interactions with AMF, it is interesting to see that CO4 acts as a main inhibitor of immune responses in this plant species. It is possible, therefore, that CO4 plays an early role to inhibit grapevine immunity to allow colonization by AMF. Interestingly, in *A. thaliana* and other non-mycotrophic plants, perception of both LCOs and CO4 results in strong suppression of MAMP-triggered immunity (Liang *et al.*, 2013; Wang *et al.*, 2023) although they do not form symbiosis either with rhizobia or with AMF. It is therefore possible that recognition of short-chain chitin oligomers and consequently the modulation of plant immunity is a mechanism as old as the first appearance of AMF ∼450 million years ago (Delaux *et al.*, 2013) which was followed later by independent differentiation in several lineages such as the *Brassicaceae* (Vigneron *et al.*, 2018) and/or for legume–rhizobia symbiosis. In addition, recent studies have demonstrated that not only are LCOs conserved molecules of symbiotic microorganisms but it is likely that all fungal microorganisms, from symbiotic to pathogenic ones, can produce them (Rush *et al.*, 2020). So, inhibition of plant defenses by LCOs and short chitin oligomers might represent an opportunity for pathogens to hijack plant immunity and promote infection. It is thus plausible that such a property was a counterselected mechanism in the never-ending co-evolution process between plants and microorganisms.

### Short-chain oligomers subvert chitin-triggered grapevine immunity to promote fungal pathogen infection

The discovery that LCOs are also produced by pathogenic fungi (Rush *et al.*, 2020) questioned the binary vision in which MAMPs are categorized in two distinct groups based on the response triggered. On the one hand, there are MAMPs that can be considered as elicitors of plant immunity and, on the other, MAMPs triggering the symbiosis through inhibition of immune responses and activation of the common symbiotic signaling pathway (CSSP). Here, we confirmed that this separation is not representative of all plant–microorganism interactions. We indeed showed that CO4 is a MAMP that can also be involved during a plant–pathogen interaction. CO4 does inhibit grapevine immunity and this inhibition directly promotes *B. cinerea* infection, which increases by >50% (Fig. 4B). Additionally, we could observe the same tendency during *E. necator* infection (Fig. 4C). Surprisingly, no effect was shown during *P. viticola* infection (Fig. 4A). *Plasmopara viticola* is an oomycete, and its cell wall is mainly composed of cellulose, glucans, and only small amounts of chitin and hydroxyproline are present (<1.5%), differing from the true fungi such as *B. cinerea* (Bakshi *et al.*, 2001; Koledenkova *et al.*, 2022). Oomycetes can largely be divided into two taxonomic groups: Peronosporomycetes and Saprolegniomycetes (Klinter *et al.*, 2019). *Plasmopara viticola* is a member of the Peronosporomycetes which has also been described as the only group devoid of GlcNAc, the main component of chitin (Mélida *et al.*, 2013). As demonstrated in this work, small fragments of chitin such as CO4 are inhibitors of grapevine immunity and in particular of the expression of a chitinase gene. The lack of GlcNAc in the *P. viticola* cell wall could explain why we did not observe any effect on this sporulation of this pathogen despite a pre-treatment with exogenous CO4. This suggests that CO4 could only inhibit chitin-triggered immunity. In addition, it has been demonstrated that lipophilic molecules such as ceramides act as MAMPs, activating plant immunity specifically during plant–oomycete interaction (Monjil *et al.*, 2024). Interestingly, these molecules are not detected in fungal pathogens, suggesting that plants also perceive oomycetes through non-chitinaceous molecules, thus deploying different signaling pathways during plant immunity.

Co-evolution between plants and microorganisms allowed the latter to adopt strategies to avoid plant immune recognition and counteract the defense responses (Trdá *et al*., 2015; Buscaill and Van Der Hoorn, 2021). One of the strategies used by fungal pathogens is based on the targeted degradation of released MAMPs by fungal effectors. The cucurbit powdery mildew fungus, *Podosphaera xanthii*, releases effectors with chitinase activity (EWCAs) when invading melon (*Cucumis melo*) plant cells (Martinez-Cruz *et al*., 2021). EWCA enzymes are indeed endochitinases and have chitinase activity against chitin oligomers with five or more units. The result is chitin degradation at random sites, producing oligomers of different sizes between two and four units but predominantly the latter (CO4). These chitinase-like activity effectors can thus degrade chitin oligomers into smaller fragments which could block the activation of chitin-triggered immunity. Interestingly, one of the most often detected chitin fragments was indeed CO4 which we described as an inhibitor of grapevine immunity due to its role in promoting *B. cinerea* infection. In addition, it has been demonstrated that *B. cinerea* possesses a specific putative group A chitinase gene, *BcchiA*, which is up-regulated in the presence of exogenous chitin (Choquer *et al.*, 2007), suggesting that this necrotrophic fungus might be able to produce shorter chitin oligomers to counteract grapevine immunity. CO4 has indeed already been identified in exudates from germinated spores of the pathogenic fungus *Colletotrichum trifolii* at similar concentrations to those in AMF exudates (Genre *et al.*, 2013). Moreover, fungal pathogens release other effectors that directly inhibit the activity of host chitinases. For example, in cotton, *Verticillium dahlia* prevents the release of immunogenic chitin fragments by Secreting Serine Protease 1 (SSEP1), which inactivates host-secreted chitinase Chi28 (Han *et al.*, 2019). Interestingly, we found that CO4 is able to down-regulate the expression level of a chitinase-encoding gene in grapevine leaves. It is plausible therefore that *B. cinerea* might activate some endo-chitinase or some chitinase-like activity effectors which degrade long chitin oligomers into shorter fragments such as CO4 which actively inhibit the expression of the host defense genes, preventing chitin-triggered immunity.

### How does grapevine distinguish symbiotic microorganisms from pathogenic ones?

MAMPs are perceived and recognized by pattern recognition receptors (PRRs) which are either surface-localized receptor-like kinases (RLKs) or receptor-like proteins (RLPs). A specific class of RLKs carries extracellular lysine motif (LysM) domains capable of binding carbohydrate-based ligands, such as fungal chitin and derivates (Couto and Zipfel, 2016; Desaki *et al.*, 2018). They often work in complex to strategically perceive different MAMPs and activate a specific response (Cao *et al.*, 2014; Zhang *et al.*, 2021; Roudaire *et al.*, 2023). In grapevine, three of the 16 LysM-RLKs identified have been characterized: VvLYK1-1 and VvLYK1-2 are the orthologs of CERK1 (Brulé *et al.*, 2019) whereas VvLYK5-1 forms a complex with VvLYK1-1 for chitin perception to activate grapevine immune responses (Roudaire *et al.*, 2023). Here we demonstrated that short chitin oligomers act as inhibitors of plant immunity in both roots and leaves of grapevine, suggesting that grapevine possesses one or more receptors for short chitin oligomers. It has been shown that in rice, the receptor OsMYR1 competes with OsCEBiP to form a complex with OsCERK1, the ortholog of VvLYK1-1, to activate either the symbiotic response or the immune response (Zhang *et al.*, 2021). Therefore, it is possible that in grapevine, a CO4 receptor could compete with VvLYK5-1 to form a new complex with VvLYK1-1, thereby blocking the immune responses of the plant and promoting the symbiotic interaction. On the other hand, when grapevine faces a pathogen such as *B. cinerea*, CO4 that might be released by the latter will act as an antagonistic MAMP, saturating the chitin-binding pockets in the extracellular domain of chitin receptors, preventing receptor complex formation, and therefore hampering the subsequent immune signaling to promote pathogen infection (Gubaeva *et al.*, 2018).

## Conclusion

Altogether, these results indicate that grapevine modulates its immune responses to stop or promote microorganism interaction, depending on the MAMP perceived. In addition, these findings impose a paradigm shift in our understanding of the role of MAMPs. Until now, CO4 was considered to be produced and used by plant microbial symbionts to promote beneficial mutual interaction. Here we show that CO4, considered for a long time as a mycorrhization factor, can also promote pathogenic infections. In addition, Rush *et al.* (2020) demonstrated that LCOs, considered as Nod and Myc factors, can be produced and used by pathogenic fungi, and CO4 was found in exudates of the pathogenic fungus *C. trifolii* (Genre *et al.*, 2013). All these results open up a new chapter in studying plant–microorganism interactions and suggest that there is no clear categorization between ‘symbiotic MAMPs’ and ‘pathogenic MAMPs’ but, on the contrary, recognition of different microorganisms relies on more complex molecular signaling. Understanding the intricate interactions between MAMPs and plant immune systems not only provides insights into fundamental plant biology but also has practical implications for agriculture. Enhancing crop resistance through the manipulation of PRRs or the engineering of robust immune responses can lead to the development of disease-resistant plant varieties, contributing to sustainable agricultural practices and food security.

## Supplementary Material

eraf247_Supplementary_Data

## Data Availability

All data supporting the findings of this study are available within the paper and its Supplementary data published online, and the materials of this study are available from the corresponding author upon reasonable request.

## Supplementary data

The following supplementary data are available at *JXB*  online.

Fig. S1. Relative expression of the defense-related gene *VvCHIT4C* after 3 h of chitin elicitation in grapevine cell suspension with or without a 30 min CO4 or LCO pre-treatment.

Fig. S2. Relative expression of the defense-related genes *VvPAL*, *VvSTS1.2*, *VvLOX9*, *VvRBOHD*, *VvJAZ*, and *VvCHIT4C* after 1 h and 3 h of chitin elicitation in grapevine roots with or without a 30 min CO4 pre-treatment.

Fig. S3. Relative expression of *VvPAL*, *VvPR1*, *VvJAZ*, *VvLOX9*, and *VvPR2* after 3, 9, and 24 h of chitin elicitation in grapevine leaves with or without a 48 h pre-treatment with CO4.

Fig. S4. Chitotetraose (CO4) effect on *E. necator* infection.

Table S1. List of primers used in this study.
