# Supplementary material for: The short chitooligosaccharide CO4 inhibits chitin-triggered immunity in grapevine and promotes the infection by Botrytis cinerea but not Plasmopara viticola
Source: J Exp Bot. 2025 Jun 4;76(16):4696–708. doi: 10.1093/jxb/eraf247 (PMC12509881; doi:10.1093/jxb/eraf247)
Supplement: eraf247_Supplementary_Data [file eraf247_supplementary_data.zip › jexbot315175-file001.pdf]

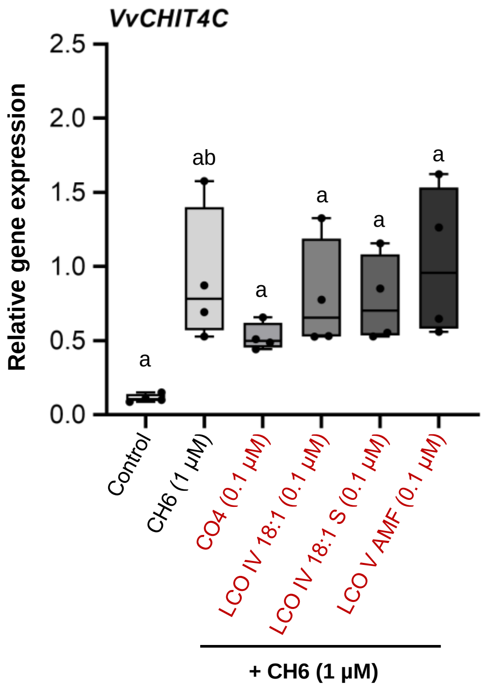

Supplementary Fig. S1. Relative expression of the defense-related genes *VvCHIT4C* after 3h of chitin elicitation in grapevine cell suspension with or without a 30 min-CO4 or -LCO pre-treatment. Relative expression of the defense-related gene *VvCHIT4C* measured by qRT-PCR 3h after chitin elicitation following a 30 min pre-treatment with CO4 or the different LCOs, as described in Fig. 1A. Boxplots represent the distribution of four independent biological repeats (n=4). Means of technical duplicates (efficiency-weighted Cq(w) values) were normalized using mean Cq(w) data of two housekeeping genes (*VvVSP54 and VvRPL18B*) before being analyzed. Different letters indicate statistically significant differences between treatments using a Kruskal-Wallis multiple comparison test followed by a “Bonferroni test” correction , $P < 0.05$).

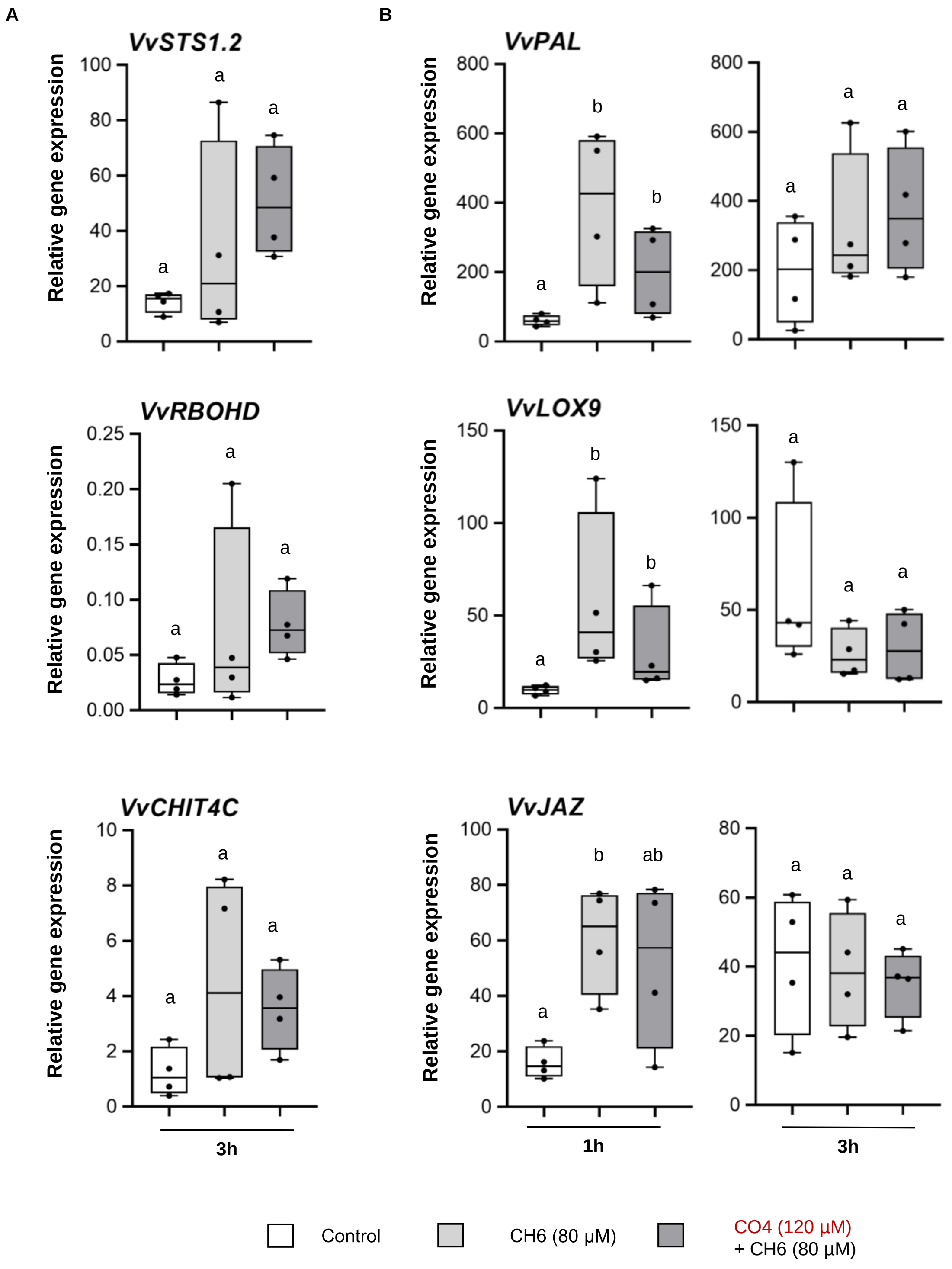

Supplementary Fig. S2. Relative expression of the defense-related genes *VvPAL, VvSTS1.2, VvLOX9, VvRBOHD, VvJAZ* and *VvCHIT4C* after 1h and 3h of chitin elicitation in grapevine roots with or without a 30 min-CO4 pre-treatment. (A) Relative expression of the defense-related genes *VvSTS1.2, VvRBOHD and VvCHIT4C* measured by qRT-PCR 3h after chitin elicitation (80 µM CH6) following a 30 min pre-treatment with CO4 (120 µM). **(B)** Relative expression of the defense-related genes *VvPAL, VvLOX9* and *VvJAZ* measured by qRT-PCR 1h and 3h after chitin elicitation (80 µM CH6) following a 30 min pre-treatment with CO4 (120 µM). Boxplots represent the distribution between four independent biological repeats (n=4) for (A) and (B). Means of technical duplicates (efficiency-weighted Cq(w) values) were normalized using mean Cq(w) data of two housekeeping genes (*VvVATP16* and *VvEF1α*) before being analyzed. Different letters indicate statistically significant differences between treatments using a Kruskal-Wallis multiple comparison test followed by "Benjamini-Hochberg test" correction ($P < 0.05$).

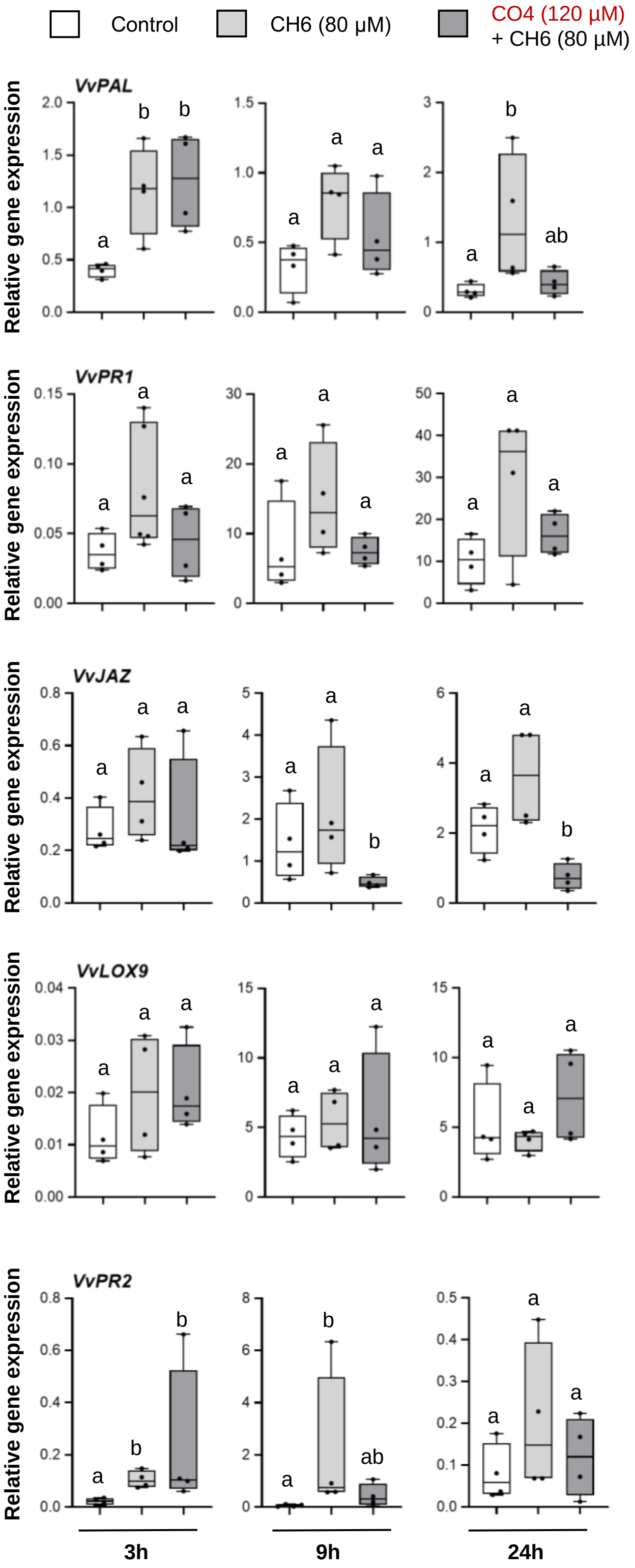

Supplementary Fig. S3. Relative expression of *VvPAL, VvPR1, VvJAZ*, *VvLOX9* and *VvPR2* after 3h, 9h and 24h of chitin elicitation in grapevine leaves with or without a 48h pre-treatment with CO4. Relative expression of *VvPAL,VvPR1, VvJAZ, VvLOX9 and VvPR2* was measured by qRT-PCR 3h, 9h and 24h after chitin elicitation (80 µM CH6) following a 48h pre-treatment with CO4 (120 µM). Boxplots represent the distribution between four independent biological repeats (n=4). Means of technical duplicates (efficiency-weighted Cq(w) values) were normalized using mean Cq(w) data of two housekeeping genes (*VvVATP16* and *VvEF1α*) before being analyzed. Different letters indicate statistically significant differences between treatments using a Kruskal-Wallis multiple comparison test followed by “Benjamini-Hochberg test” correction (P< 0.05). and

	RB	CT ratio *E. necator / V. vinifera*	Symptoms observation
Ctrl	1	1	9
E. necator	1	3358,41	6,33
E. necator + CO4	1	4276,07	5
Ctrl	2	1	9
E. necator	2	1002,53	4,34
E. necator + CO4	2	1205,39	3,67
Ctrl	3	1	9
E. necator	3	5741,46	5
E. necator + CO4	3	7852,18	5

Supplementary Fig. S4. Chitotetraose (CO4) effect on *E. necator* infection. (A) Symptom assessment (OIV scale) of *E. necator* in control plants (Ctrl), plants inoculated with *E. necator,* and plants inoculated with *E. necator* after treatment with 120 µM CO4. The OIV scale ranges from 9 (no infection) to 1 (severe infection).

Supplementary Table S1. List of primers used in this study.

Gene	ID	Sequence (5' → 3')
VvRPL18B	VvRPL18B_qF	CCCCTATGCTTTTTGTGGACTTG
VvRPL18B	VvRPL18B_qR	TGCTCGTTTGGGACAATAAACC
VvVATP16	VvVATP16_qF	CTTCTCCTGTATGGGAGCTG
VvVATP16	VvVATP16_qR	CCATAACAACTGGTACAATCGAC
VvVPS54	VvVPS54_qF	GCTGTTTTTGCGGCTTGTGA
VvVPS54	VvVPS54_qR	ACCTTCCACCAATCTTCTCCGT
VvEF1α	Vv_EF1α_qF	GAACTGGGTGCTTGATAGGC
VvEF1α	Vv_EF1α_qR	AACCAAAATATCCGGAGTAAAAGA
VvCHIT4C	VvCHIT4C_qF	GCAACCGATGTTGACATATCA
VvCHIT4C	VvCHIT4C_qR	CGTCGCCCTAGCAAGTGAG
VvSTS1.2	VvSTS1.2_qF	AGGAAGCAGCATTGAAGGCTC
VvSTS1.2	VvSTS1.2_qR	TGCACCAGGCATTTCTACACC
VvRBOHD	VvRBOHD_qF	CACCACCATGCTTCAGTCCCTCCAT
VvRBOHD	VvRBOHD_qR	AGCGATCTTCTTGAAGACTTGTCGCC
VvJAZ	VvJAZ_qF	GGCGAGGGGACCGGAGAAGT
VvJAZ	VvJAZ_qR	TCGGGCGTGCCGTTTCCTTC
VvPR1	VvPR1_qF	GCGTGGGTGGGGAATGCCGA
VvPR1	VvPR1_qR	GATGTTGTCCCTGATAGTTGC
VvPR2	VvPR2_qF	TCAGCCGTCCTCGGCAAATCA
VvPR2	VvPR2_qR	TTGGCCAGGAGTGGGGAGCC
VvLOX9	VvLOX9_qF	CTGCGTGGCTTCTGCTCTC
VvLOX9	VvLOX9_qR	CCATCAATCTGCGGCTTATC
VvPAL	VvPAL_qF	ACTCTCCATCGACAACACCCG
VvPAL	VvPAL_qR	TGCTCACCACTTTCGACATGG
VvEN	VvEN_qF	CTTCGGATTTTTGGGTCAGA
VvEN	VvEN_qR	GGCAGATCATTGGATTCTT
